# The Therapeutic Potential of PLGA Nanoparticles in Alzheimer's Disease

**DOI:** 10.1002/alz70859_105801

**Published:** 2025-12-25

**Authors:** Bryan P Sanders, Igal Ifergan

**Affiliations:** ^1^ University of Cincinnati, Cincinnati, OH USA

## Abstract

**Background:**

We test the therapeutic potential of FDA approved immunoregulatory PLGA nanoparticles in C57BL6/SJLJ/5xFAD mice

**Method:**

C57BL6/SJLJ/5xFAD mice were treated with PLGA nanoparticles, and behavioral analysis and flow cytometry were used to determine cognitive performance and the inflammatory phenotypes of myeloid cells in the CNS after treatment.

**Result:**

At both 3 weeks and 2 months post a 4‐week treatment with PLGA, the behavioral deficits in 5xFAD mice were restored to the same level as age matched wild‐type mice in the Elevated Plus Maze. Phenotypic analysis of cells in the CNS revealed a significant decrease in microglia and infiltrating myeloid cell activation and inflammatory markers in PLGA‐treated mice. Importantly, we observed a significant decrease in DAM proportion in PLGA‐treated mice, which showed a significant decrease in TNF‐a expression.

**Conclusion:**

FDA approved PLGA nanoparticles infiltrate the CNS in 5xFAD mice and induce an immunoregulatory effect on myeloid cells, as well as behavioral phenotypes which mimic aged matched wild‐type mice, suggesting that nanoparticle based immunotherapy could a be a promising strategy in treating Alzheimer's disease.

